# Supplementary material for: BUB-1 promotes amphitelic chromosome biorientation via multiple activities at the kinetochore
Source: eLife. 2018 Dec 14;7:e40690. doi: 10.7554/eLife.40690 (PMC6303103; doi:10.7554/eLife.40690)
Supplement: Supplementary file 1. — (A) List of worm strains used in this study. (B) List of templates and primers used to synthesize double stranded RNA. (C) Targeted regions for CRISPR-Cas9 mediated hcp-1CENP-F and hcp-2CENP-F deletion mutant generation. (D) Images and datasets presented in different figures and panels. [file elife-40690-supp1.docx]

**SUPPLEMENTARY TABLES**

**Supplementary file 1A. *C. elegans* strains used**

| **Strain** | **Genotype** |
| --- | --- |
| N2 | *Wild Type (Ancestral N2 Bristol)* |
| JDU221 | *ItSi1[pOD809/pJE110; Pknl-1::Knl-1reencoded::mCherry; cb-unc-119(+)]II; ddIs6[GFP::tbg-1; unc-119(+)]V; unc-119(ed3)III?** |
| OD2207 | *ltSi683[pOD1873; 5'CenpA_GFP_Cpar1-Tail_CenpA_3'CenpA; cb-unc-119(+)]II; unc-119(ed3)III?; ltIs37 [pAA64; pie-1/mCherry::his-58; unc-119 (+)]IV** |
| JDU480 | *it75[knl-1::mCherry]III; unc-119(ed3) III?; hcp-2(ijm6)V; ddIs6[GFP::tbg-1; unc-119(+)]V** |
| JDU440 | *ltSi1[pOD809/pJE110; Pknl-1::Knl-1reencoded::mCherry; cb-unc-119(+)]II; unc-119(ed3) III?; hcp-2(ijm6)V; ddIs6[GFP::tbg-1; unc-119(+)]V** |
| JDU441 | *ltSi44[pOD1039/pJE170; Pknl-1::Knl-1reencoded(Mutant D85-505)::mCherry; cb-unc-119(+)]II; unc-119(ed3) III?; hcp-2(ijm6)V; ddIs6[GFP::tbg-1; unc-119(+)]V** |
| JDU529 | *It28[ska-1::gfp + Loxp)I; ijmSi31 [pJD446_pJD362_Mos2_Pmex-5_mCherry::his-11_3'UTRtbb-2] II; hcp-2(ijm6)V** |
| JDU498 | *ltSi268[pOD/pTK013; Pbub-1::Bub1reencoded; cb-unc-119(+)]II; unc-119(ed3) III?; it75[knl-1::mCherry]III; hcp-2(ijm6)V; ddIs6[GFP::tbg-1; unc-119(+)]V** |
| JDU533 | *ska-1(It29[loxp])I; it75[knl-1::mCherry]III; unc-119(ed3) III?; hcp-2(ijm6)V; ddIs6[GFP::tbg-1; unc-119(+)]V** |
| PHX441 | *ijmSi31[pJD446_pJD362_Mos2_Pmex-5_mCherry::his-11_3'UTRtbb-2]II; unc-119(ed3) III?; hcp-1(syb441[GFP ::HCP-1])V** |
| PHX444 | *ijmSi31[pJD446_pJD362_Mos2_Pmex-5_mCherry::his-11_3'UTRtbb-2]II; unc-119(ed3) III?; hcp-2(syb444[GFP ::HCP-2])V** |
| JDU146 | *ijmSi3 [pJD342/pJD330; ChrI_5'mex-5_cls-2reenc::GFP_tbb-2; cb-unc-119 (+)]I; ijmSi31 [pJD446_pJD362_Mos2_Pmex-5_mCherry::his11_3'UTRtbb-2]II; unc-119(ed3) III?** |
| JDU494 | *ltSi270[pOD/pTK014; Pbub-1::Bub-1reencoded(mutantK718R, D847N); cb-unc-119(+)]II; unc-119(ed3) III?; it75[knl-1::mCherry]III; unc-119(ed3) III?; hcp-2(ijm6)V; ddIs6[GFP::tbg-1; unc-119(+)]V** |
| JDU495 | *ltSi264[pOD/pTK; Pbub-1::Bub-1reencoded(mutantD814N); cb-unc-119(+)]II; unc-119(ed3) III?; it75[knl-1::mCherry]III; hcp-2(ijm6)V; ddIs6[GFP::tbg-1; unc-119(+)]V** |
| JDU513 | *ijmSi112[pJD669_Mos2_Pbub-1::Bub-1reencoded(mutantDeltaKD)::mCherry; cb-unc-119(+)]II; it75[knl-1::mCherry]III; unc-119(ed3) III?; hcp-2(ijm6)V; ddIs6[GFP::tbg-1; unc-119(+)]V** |
| OD1870 | *ItSi1003[pTK048; Pmex-5::zwl-1::gfp::tbb-2_3'UTR::operon linker::mCherry::his-58::tbb-2_3'UTR; cb-unc-119(+)]I* |
| JDU529 | *It28[ska-1::gfp + Loxp)I; ijmSi31 [pJD446_pJD362_Mos2_Pmex-5_mCherry::his-11_3'UTRtbb-2] II; unc-119(ed3) III?; hcp-2(ijm6)V** |
| JDU540 | *ska-1(It29[loxp])I; ltSi270[pOD/pTK014; Pbub-1::Bub1reencoded(mutantK718R, D847N); cb-unc-119(+)]II; unc-119(ed3) III?; it75[knl-1::mCherry]III; hcp-2(ijm6)V; ddIs6[GFP::tbg-1; unc-119(+)]V** |
| JDU541 | *ska-1(It29[loxp])I; ijmSi112[pJD669_Mos2_Pbub-1::Bub-1reencoded(mutantDeltaKD)::mCherry; cb-unc-119(+)]II; it75[knl-1::mCherry]III; unc-119(ed3) III?; hcp-2(ijm6)V; ddIs6[GFP::tbg-1; unc-119(+)]V** |
| JDU55 | *ijmSi13 [pJD406/pJD342; ChrI_5'mex-5_bub-1::sfGFP; cb-unc-119(+)]I; unc-119(ed3)III?; ltIs37 [pAA64; pie-1/mCherry::his-58; unc-119 (+)]IV** |
| JDU258 | *ijmSi31 [pJD446_pJD362_Mos2_Pmex-5_mCherry::his-11_3'UTRtbb-2] II; unc-119(ed3) III?; hcp-1(ijm4)V** |
| JDU302 | *hcp-2(ijm6)V* |
| JDU344 | *ijmSi31 [pJD446_pJD362_Mos2_Pmex-5_mCherry::his-11_3'UTRtbb-2] II; unc-119(ed3) III?; hcp-2(ijm6)V** |
| JDU69 | *ijmSi13 [pJD406/pJD342; ChrI_5'mex-5_bub-1::sfGFP; cb-unc-119(+)]I; ltSi1[pOD809/pJE110; Pknl-1::KNL-1reencoded::mCherry; cb-unc-119(+)]II; unc-119(ed3) III ?** |

| JDU71 | *ijmSi13 [pJD406/pJD342; ChrI_5'mex-5_bub-1::sfGFP; cb-unc-119(+)]I; ltSi44[pOD1039/pJE170; Pknl-1::KNL-1reencoded(Mutant D85-505)::mCherry; cb-unc-119(+)]II; unc-119(ed3) III ?** |
| --- | --- |
| JDU326 | *ltSi259[pOD/pTK007; Phcp-1::GFP::Hcp1 reencoded; cb-unc-119(+)]I; ltSi264[pOD/pTK011; Pbub-1::Bub1 reencoded ::mCherry; cb-unc-119(+)]II; unc-119(ed3) III?** |
| JDU327 | *ltSi259[pOD/pTK007; Phcp-1::GFP::Hcp1 reencoded; cb-unc-119(+)]I; ltSi266[pOD/pTK012; Pbub-1::Bub1 reencoded(mutantK718R, D847N)::mCherry; cb-unc-119(+)]II; unc-119(ed3) III?** |
| JDU329 | *ltSi259[pOD/pTK007; Phcp-1::GFP::Hcp1 reencoded; cb-unc-119(+)]I; ltSi758[pOD1966/pTK028; Pbub-1::Bub1reencoded(mutantD814N)::mCherry; cb-unc-119(+)]II; unc-119(ed3) III?* |
| JDU507 | *ltSi259[pOD/pTK007; Phcp-1::GFP::Hcp1 reencoded; cb-unc-119(+)]I; ijmSi112[pJD669_Mos2_Pbub-1::BUB-1reencoded(mutantDeltaKD)::mCherry; cb-unc-119(+)]II; unc-119(ed3)III?* |
| JDU538 | *ijmSi31[pJD446_pJD362_Mos2_Pmex-5_mCherry::his-11_3'UTRtbb-2]II; dnc-2[prt42(N-terminal 3xflag::GFP)]III; unc-119(ed3) III?; hcp-2(ijm6)V** |
| JCC504 | *air-2(or207ts) I; transgene ojIs1 [pie-1::gfp-beta-tbb-2] III; unc-119(ed3) III?; ltIs37 [pAA64; pie-1/mCherry::his-58; unc-119 (+)] IV** |
| JDU552 | *ltSi264[pOD/pTK011; Pbub-1::Bub1reencoded::mCherry; cb-unc-119(+)]II; dnc-2[prt42(N-terminal 3xflag::GFP)]III; unc-119(ed3) III?; hcp-2(ijm6)V** |
| JDU553 | *ijmSi112[pJD669_Mos2_Pbub-1::Bub-1reencoded(mutantDeltaKD)::mCherry; cb-unc-119(+)]II; dnc-2[prt42(N-terminal 3xflag::GFP)]III; unc-119(ed3)III?; hcp-2(ijm6)V** |
| OD987 | *ltSi264[pOD/pTK011; Pbub-1::Bub1 reencoded ::mCherry; cb-unc-119(+)]II; unc-119(ed3) III* |
| OD989 | *ltSi266[pOD/pTK012; Pbub-1::Bub1 reencoded(mutantK718R, D847N)::mCherry; cb-unc-119(+)]II; unc-119(ed3) III* |
| JDU500 | *ijmSi112[pJD669_Mos2_Pbub-1::BUB-1reencoded(mutantDeltaKD)::mCherry; cb-unc-119(+)]II; unc-119(ed3)III* |
| JDU548 | *ltSi264[pOD/pTK011; Pbub-1::Bub1 reencoded ::mCherry; cb-unc-119(+)]II; unc-119(ed3)III?** |
| JDU544 | *It28[ska-1::gfp + Loxp)I; ijmSi112[pJD669_Mos2_Pbub-1::BUB-1reencoded(mutantDeltaKD)::mCherry; cb-unc-119(+)]II; unc-119(ed3)III ?** |
| JDU545 | *It28[ska-1::gfp + Loxp)I; ltSi266[pOD/pTK012; Pbub-1::Bub1 reencoded(mutantK718R, D847N)::mCherry; cb-unc-119(+)]II; unc-119(ed3)III** |

*[*unc-119(ed3)?*] was present in the parental strains, but these strains have not been directly sequenced to determine if the *unc-119* gene contains the *ed3* mutation.

**Supplementary file 1B. Templates and primers used to synthesise *dsRNA***

| **Gene** | **Primer 1*** | **Primer 2*** | **Template** | **Final [C] (μg/μl)** |
| --- | --- | --- | --- | --- |
| R06C7.8  *(bub-1)* | 5’-aattaaccctcactaaagg  AGCAAATCAAGCCGTTCAGC-3’ | 5’-taatacgactcactatagg  AGCATCGCGAAGAATGTTGC-3’ | N2 genomic DNA | 1,9 |
| ZK1055.1  *(hcp-1)* | 5’-aattaaccctcactaaagg  aagcgccagcaaaccgagtcgcC-3’ | 5’-taatacgactcactatagg  gtcaatgtgacctttgacaggaagc-3’ | N2 genomic DNA | 1,25 |
| T06E4.1  *(hcp-2)* | 5’-aattaaccctcactaaagg  tcgttgtctccaattccacA-3’ | 5’-taatacgactcactatagg  tctcggaaaggaatcgaaaA-3’ | N2 genomic DNA | 2,1 |
| R107.6  *(cls-2)* | 5’-aattaaccctcactaaagg  ACGTGCGAATGTGAACTGGACG-3’ | 5’-taatacgactcactatagg  TCAACAACGCATCTTATTGACG-3’ | *cls-2* cDNA  (pJD33) | 1,6 |
| C02F5.1  *(knl-1)* | 5’-aattaaccctcactaaagg  aatctcgaatcaccgaaatgtC-3’ | 5’-taatacgactcactatagg  ttcacaaacttggaagccgctG-3’ | N2 genomic DNA | 1,6 |
| R06C7.8  *(bub-1)* | 5’-aattaaccctcactaaagg  ggataattttatgatcaccag-3’ | 5’-taatacgactcactatagg  ctacttttggttggcggcaag-3’ | *bub-1* cDNA  (pJD42) | 1 |
| R12B2.4  *(him-10)* | 5’-aattaaccctcactaaagg  attctggcaacaagctggac-3’ | 5’-taatacgactcactatagg  acgctgacgctcttcacttT-3’ | N2 genomic DNA | 1,6 |
| Y39G10AR.2  *(zwl-1)* | 5’-aattaaccctcactaaagg  atgccactcaccatcgagcaG-3’ | 5’-taatacgactcactatagg  ggatcagtgaagcgagatgactc-3’ | N2 cDNA | 1,5 |
| C06A8.5  *(spdl-1)* | 5’-aattaaccctcactaaagg  AACGTTACCCGAATGACCAC-3’ | 5’-taatacgactcactatagg  CCTAATTGAGGCATGGGTTC-3’ | N2 genomic DNA | 1,2 |
| F59E12.2  *(zyg-1)* | 5’- aattaaccctcactaaagg  TGGACGGAAATTCAAACGAT -3’ | 5’- taatacgactcactatagg  AACGAAATTCCCTTGAGCTG -3’ | N2 cDNA | 1,4 |
| C50F4.11  *(mdf-1)* | 5’- aattaaccctcactaaagg  AGCATCCTCAAGTCGTTCGT -3’ | 5’- taatacgactcactatagg  AAGCGAAGTTGGCTGAAAAA -3’ | N2 genomic DNA | 1,3 |

*Lowercase letters denote T3 and T7 sequences included for RNA synthesis­­

**Supplementary file 1C. Targeted regions for CRISPR-Cas9 mediated *hcp-1* and *hcp-2* mutant generation**

| **Sequence targeted by sgRNA** | **gene** | **Theoretical cut position** |
| --- | --- | --- |
| 5’-GTAGTCCTCAAAATTCGACA-3’ | *hcp-1* | 723 bp upstream of ATG |
| 5’-GGTTCAAGAAAGCGACGAGA-3’ | *hcp-1* | 466 bp downstream of ATG |
| 5’-aattgtggcagagaagacga-3’ | *hcp-2* | 1046 bp upstream of ATG |
| 5’-ATACTCCGTCAACACCACTT-3’ | *hcp-2* | 540 bp downstream of ATG |

**Supplementary file 1D. Images and datasets presented in different figures and panels**

| **Condition** | **Kymograph*** | **Sister segregation images** | **Fluorescence images**** | **Pole to Pole distances** | **Chromosome span** | **Chromosome segregation behaviour** | **Fluorescence intensity** | **n embryos analyzed** |
| --- | --- | --- | --- | --- | --- | --- | --- | --- |
| JDU221 Control | 1B |  |  | 1C, 1S2C | 1C, 1S2C |  |  | 19 |
| JDU221 *bub-1(RNAi)* | 1B |  |  | 1C | 1C |  |  | 22 |
| JDU221 *hcp-1/2(RNAi)* | 1B |  |  | 1C, 1S2C | 1C, 1S2C |  |  | 22 |
| JDU221 *cls-2(RNAi)* | 1B |  |  | 1C | 1C |  |  | 15 |
| JDU221 *cls-2+bub-1(RNAi)* | 1B |  |  | 1C | 1C |  |  | 11 |
| JDU440 *hcp-1+bub-1(RNAi)* | 1B, 4C | 1G |  | 1C, 4D | 1C, 4D | 1F, 4E |  | 11 |
| PHX441 |  |  | 1E, 1S1A |  |  |  | 1E, 1S1B | 12 |
| PHX441 *bub-1(RNAi)* |  |  | 1E, 1S1A |  |  |  | 1E, 1S1B | 8 |
| PHX444 |  |  | 1E, 1S1A |  |  |  | 1E, 1S1B | 7 |
| PHX444 *bub-1(RNAi)* |  |  | 1E, 1S1A |  |  |  | 1E, 1S1B | 8 |
| JDU146 |  |  | 1E, 1S1A |  |  |  | 1E, 1S1B, 6D | 12 |
| JDU146  *bub-1(RNAi)* |  |  | 1E, 1S1A |  |  |  | 1E, 1S1B | 11 |
| JDU440 Control | 1S2B, 5S2D | 1G |  | 1S2C, 5S2E | 1S2C, 5S2E | 1H, 4E |  | 12 |
| JDU440 *bub-1(RNAi)* |  | 1G |  |  |  | 1H, 4E |  | 23 |
| JDU440  *hcp-1(RNAi)* | 4C 1S2B | 1G |  | 4D, 1S2C | 4D, 1S2C | 1H, 4E |  | 14 |
| JDU440 *knl-1(RNAi)* | 2B | 2S1B |  | 2C | 2C | 2D |  | 12 |
| JDU440 *hcp-1+knl-1(RNAi)* | 2B | 2S1B |  | 2C | 2C | 2D |  | 11 |
| JDU441 *knl-1(RNAi)* | 2B | 2S1B |  | 2C | 2C | 2D |  | 15 |
| JDU441 *hcp-1+knl-1(RNAi)* | 2B | 2S1B |  | 2C | 2C | 2D |  | 16 |
| JDU498  *bub-1(RNAi)* | 3B, 3S1A | 3S1E |  | 3C, 3S1B | 3C, 3S1B | 3D, 3S1D |  | 15 |
| JDU498  *hcp-1+bub-1(RNAi)* | 3B, 5C | 3S1E |  | 3C, 5E | 3C, 5E | 3D, 5D |  | 12 |
| JDU494  *hcp-1+bub-1(RNAi)* | 3B, 5C | 3S1E |  | 3C, 5E | 3C, 5E | 3D, 5D |  | 13 |
| JDU495  *hcp-1+bub-1(RNAi)* | 3B | 3S1E |  | 3C | 3C | 3D |  | 14 |
| JDU513  *hcp-1+bub-1(RNAi)* | 3B, 5C | 3S1E |  | 3C, 5E | 3C, 5E | 3D, 5D |  | 9 |
| JDU440  *hcp-1+zwl-1(RNAi)* | 4C | 4S1E |  | 4D | 4D | 4E |  | 13 |
| JDU440 *zwl-1(RNAi)* |  |  |  |  |  | 4E |  | 12 |
| OD1870 Control |  |  | 4B, 4S1A |  |  |  | 4B, 4S1B | 11 |
| OD1870  *bub-1(RNAi)* |  |  | 4B, 4S1A |  |  |  | 4B, 4S1B | 9 |
| OD1870 *hcp-1/2(RNAi)* |  |  | 4B, 4S1A |  |  |  | 4B, 4S1B | 9 |
| JDU538 Control |  |  | 4B, 4S1C |  |  |  | 4B, 6D, 4S1D | 6 |
| JDU538  *bub-1(RNAi)* |  |  | 4B, 4S1C |  |  |  | 4B, 4S1D | 6 |
| JDU538 *hcp-1(RNAi)* |  |  | 4B, 4S1C |  |  |  | 4B, 4S1D | 8 |
| JDU529 Control |  |  | 5B, 5S2A |  |  |  | 5B, 6D, 5S2B | 11 |
| JDU529  *hcp-1(RNAi)* |  |  | 5B, 5S2A |  |  |  | 5B, 5S2B | 9 |
| JDU529 *hcp-1+bub-1(RNAi)* |  |  | 5B, 5S2A |  |  |  | 5B, 5S2B | 12 |
| JDU529 *zyg-1(RNAi)* |  |  | 5B, 5S2A |  |  |  | 5B, 5S2C | 11 |
| JDU529 *bub-1(RNAi)* |  |  | 5S2A |  |  |  | 5S2B | 11 |
| JDU540 *hcp-1+bub-1(RNAi)* | 5C | 5S2G |  | 5E | 5E | 5D |  | 13 |
| JDU541 *hcp-1+bub-1(RNAi)* | 5C | 5S2G |  | 5E | 5E | 5D |  | 11 |
| JDU55 Control |  |  | 5S2H |  |  |  | 6D, 5S2I | 5 |
| OD2207 Control |  |  | 1S1D |  |  |  | 1S1E | 16 |
| OD2207 *bub-1(RNAi)* |  |  | 1S1D |  |  |  | 1S1E | 12 |
| OD2207 *hcp-1/2(RNAi)* |  |  | 1S1D |  |  |  | 1S1E | 17 |
| OD2207 *cls-2(RNAi)* |  |  | 1S1D |  |  |  | 1S1E | 9 |
| JDU258 Control | 1S2D |  |  |  |  |  |  | 12 |
| JDU258 *hcp-2(RNAi)* | 1S2D |  |  |  |  |  |  | 9 |
| JDU258 *hcp-2+bub-1(RNAi)* | 1S2D |  |  |  |  |  |  | 10 |
| JDU69 *knl-1(RNAi)* |  |  | 2S1A |  |  |  | 2S1A | 14 |
| JDU71 *knl-1(RNAi)* |  |  | 2S1A |  |  |  | 2S1A | 13 |
| JDU494 *bub-1(RNAi)* | 3S1A | 3S1E |  | 3S1B | 3S1B |  | 3S1D | 12 |
| JDU495 *bub-1(RNAi)* | 3S1A | 3S1E |  | 3S1B | 3S1B |  | 3S1D | 14 |
| JDU513 *bub-1(RNAi)* | 3S1A | 3S1E |  | 3S1B | 3S1B |  | 3S1D | 13 |
| JDU326 *bub-1(RNAi)* |  |  | 3S1C |  |  |  | 3S1C | 10 |
| JDU327 *bub-1(RNAi)* |  |  | 3S1C |  |  |  | 3S1C | 10 |
| JDU329 *bub-1(RNAi)* |  |  | 3S1C |  |  |  | 3S1C | 11 |
| JDU507 *bub-1(RNAi)* |  |  | 3S1C |  |  |  | 3S1C | 10 |
| JDU552 *bub-1(RNAi)* |  |  | 4S1F |  |  |  | 4S1F | 6 |
| JDU553 *bub-1(RNAi)* |  |  | 4S1F |  |  |  | 4S1F | 6 |
| JCC504 Control | 5S1B |  |  | 5S1C | 5S1C |  |  | 9 |
| JCC504 *hcp-1/2(RNAi)* | 5S1B |  |  | 5S1C | 5S1C |  |  | 18 |
| JDU533 Control | 5S2D |  |  | 5S2E | 5S2E |  |  | 11 |
| JDU548 *bub-1(RNAi)* |  |  |  |  |  |  | 5S2G | 20 |
| JDU544 *bub-1(RNAi)* |  |  |  |  |  |  | 5S2G | 11 |
| JDU545 *bub-1(RNAi)* |  |  |  |  |  |  | 5S2G | 7 |
| JDU55 *hcp-1/2(RNAi)* |  |  | 5S2H |  |  |  | 5S2I | 5 |
| JDU513 *bub-1(RNAi)* at 15°C |  | 6A |  |  |  | 6A |  | 15 |
| JDU513 *bub-1(RNAi)* at 24°C |  | 6A |  |  |  | 6A |  | 15 |

*Kymographs were generated from different representative embryos when the same condition is presented in different panels.

**The same embryo was used in cases when for a same condition, one panel represents a single timepoint, and another panel represents a temporal series.
